# Home treatment of type VI mucopolysaccharidosis (Maroteaux‐Lamy syndrome) an alternative at this time of COVID‐19 pandemic: A case in Peru

**DOI:** 10.1002/ccr3.3420

**Published:** 2020-10-31

**Authors:** Jenny Lucy Cortez Miranda, Lincoln Blacido Trujillo, María Elena Liendo Chocano

**Affiliations:** ^1^ Pediatric Service Department of Medicine Central Military Hospital Lima Peru

**Keywords:** dermatan sulfate, enzyme replacement therapy, galsulfase, glycosaminoglycan, mucopolysaccharidosis type VI

## Abstract

We report a patient with mucopolysaccharidosis type VI, on long‐term enzyme replacement home therapy. Results support the efficacy and safety benefits, with additional advantage of home therapy to minimize the risk of community‐transmitted infections.

## Graphical abstract

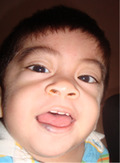

## 1 INTRODUCTION

We report a patient with mucopolysaccharidosis type VI, on long‐term (2 years, 6 months) galsulfase enzyme replacement home therapy. Results support the efficacy and safety benefits, with additional advantage of home therapy to minimize the risk of community‐transmitted infections at this time of the COVID‐19 pandemic.

Mucopolysaccharidosis type VI (MPS VI, Maroteaux‐Lamy syndrome) is an autosomal recessive lysosomal storage disorder caused by a deficiency of the N‐acetylgalactosamine 4 sulfatase enzyme (also known as arylsulfatase B or ASB), which results in the accumulation of the glycosaminoglycan (GAG) dermatan sulfate (DS) in different tissues of the body.^1^ The disease is associated with high morbidity and reduced life expectancy,^2^, ^3^ although its incidence is lower than that of other MPS (1/455 000 live births).^4^ In Monte Santo, Brazil, the prevalence is estimated at 20/1 000 000, probably as a result of a founding effect and inbreeding, with a single homozygous mutation present (p.H178L).^5^ No studies reporting the prevalence of this disease are available in Peru or other Latin American countries.

The N‐acetylgalactosamine 4 sulfatase enzyme is found predominantly not only in the skin, but also in tendons, blood vessels, airways, and heart valves.^6^ Studies have shown that DS accumulation creates an inflammatory response through the tumor necrosis factor (TNF) pathway, resulting in chondrocyte apoptosis and ensuing progressive arthropathy.^7^, ^8^ It affects bone, cartilage, liver, spleen, ligaments, joints, heart valves, airways, meninges, and corneas.

As a result of the accumulation of DS in the different tissues, clinical signs and symptoms are multisystemic and heterogenous. There is evidence of growth stunting, coarse facies, thick hair, skeletal deformities, frequent upper airway infections, hepatosplenomegaly, hearing loss, sleep apnea, and stiff joints.^2^ Other anatomical abnormalities and heart valve dysfunction have been reported in all patients.^3^

Patients with MPS VI generally appear healthy at birth, with symptoms usually manifesting in early infancy as a result of increased GAG concentration in the cells. The clinical presentation varies according to the age of onset and the rate of progression of the disease. Depending on the symptoms, the progression of MPS VI is categorized as classical or atypical.^9^ The highest urinary GAG levels are associated with rapid progression of the disease.^9^

There is a genotype‐phenotype correlation in ASB mutations according to residual enzyme activity, which is responsible for the heterogeneity of the clinical presentation.^10^ The definitive diagnosis is made on the basis of deficient ASB enzyme activity confirmed in cultured fibroblasts or isolated leukocytes (value < 10% the lower limit of normal) and/or demonstration of two mutations that cause the disease.^2^, ^3^, ^11^ Elevated GAGs in the urine and high DS concentrations are markers of disease activity but, alone, are not diagnostic methods.

Patients with MPS VI require an interdisciplinary approach to the management of their disease. Enzyme replacement therapy (ERT) with galsulfase was approved in 2005‐2006 in Europe, the United States, Brazil, Ecuador, and Australia, among other countries.^12^, ^13^ This therapy slows down disease progression as it restores ASB enzymatic function, preventing GAG accumulation.^14^ It is associated with improved functional endurance tests (6‐minute and 12‐minute walk test, and 3‐minute stair climb), reduction in urinary GAG levels, stabilization of cardiac and pulmonary progression at 5‐year follow‐up, and reduction in 10‐year mortality.^13^, ^15^, ^16^, ^17^ Even though therapy is effective, it requires weekly intravenous infusions over 3‐4 hours in a hospital setting, which is one of the drawbacks for patients and families.^18^

In developed countries, home therapy has been previously described as a safe option in MPS patients, alleviating treatment burden.^18^, ^19^, ^20^

The objective of this article was to report the case of a patient in Peru with a diagnosis of MPS VI, focusing on the general characteristics of MPS VI, the course of the disease, and findings related to the use of enzyme replacement therapy over a 6‐year period, of which, 2 years and 6 months the therapy was administered at home.

## 2 CASE HISTORY/EXAMINATION

**FIGURE 1:**
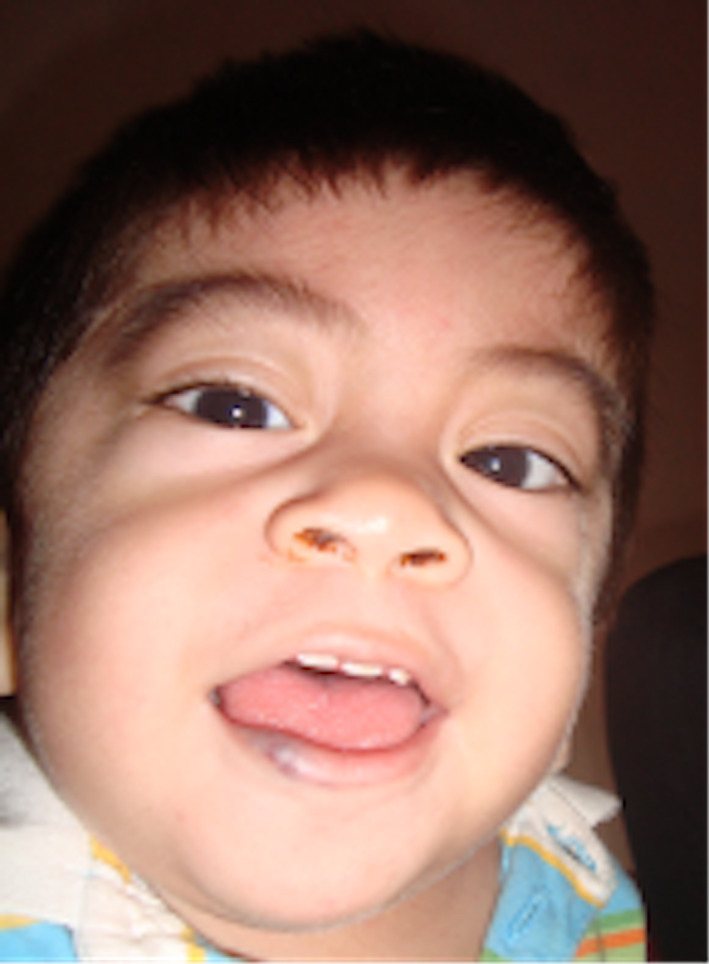
Physical appearance of the patient: The patient at 1 y of age showing evidence of coarse facies, prominent forehead, thick hair, bushy eyebrows, bulging eyes, broad nasal base, depressed nasal bridge, and macroglossia

We describe the case of a male patient aged 9 years and 10 months, coming from northern Peru, born to a primigravida, with no relevant perinatal history, who exhibited normal psychomotor development up to 6 months of age. After that time, the patient developed evidence of discrete palpable and painless lumbar spine deformation together with increased head circumference, stunting, and progressive joint stiffness affecting his ability to walk and grasp objects (1 year and 6 months of age) (Figure 1). Moreover, changes in features began to develop, with coarse facies, prominent forehead, thick hair, hirsutism, thick eyebrows, bulging eyes, broad nasal base, depressed nasal bridge, macroglossia, and small, spaced‐out teeth. Persistent and worsening lumbar deformity and joint stiffness prompted referral to a center specialized in physical medicine and rehabilitation where the patient was diagnosed with L2‐L3 hemivertebrae and subjected to surgery for bilateral hip dysplasia.

### 2.1 Investigations and treatment

At 3 years of age, the patient was seen at the genetics service of the Child's Health Specialized Institute (Instituto Especializado de Salud del Niño) where metabolic screening tests with cetyltrimethylammonium bromide and toluidine blue were positive for GAGs in the urine, and fluorometric quantification of N‐acetylgalactosamine 4 sulfatase in the blood (carried out in Germany) showed a result of 20.8 µmol/L/h (normal reference values >50 µmol/L/h).

The *ASB* gene was analyzed using the mass sequencing technique based on amplicon sequencing at Centogene, Rostock, Germany (NM_000046.3). Sequencing revealed a homozygous deletion covering exons 7 and 8. Quantitative PCR assay was performed (NM_000046.3) with eight specific amplicons covering exons 1 to 8 (or part of them). Possible splicing effects were evaluated using prediction tools for variants in splicing regions. This deletion had been previously identified in two patients affected with compound heterozygosity and a second pathogenic variant, according to the Centogene internal database (unpublished data). The patient's biochemical and genetic results were classified as pathogenic (class 1) in accordance with the recommendations of Centogene and the American College of Medical Genetics and Genomics (ACMG). ASB gene could not be investigated in the patient's parents.

**FIGURE 2:**
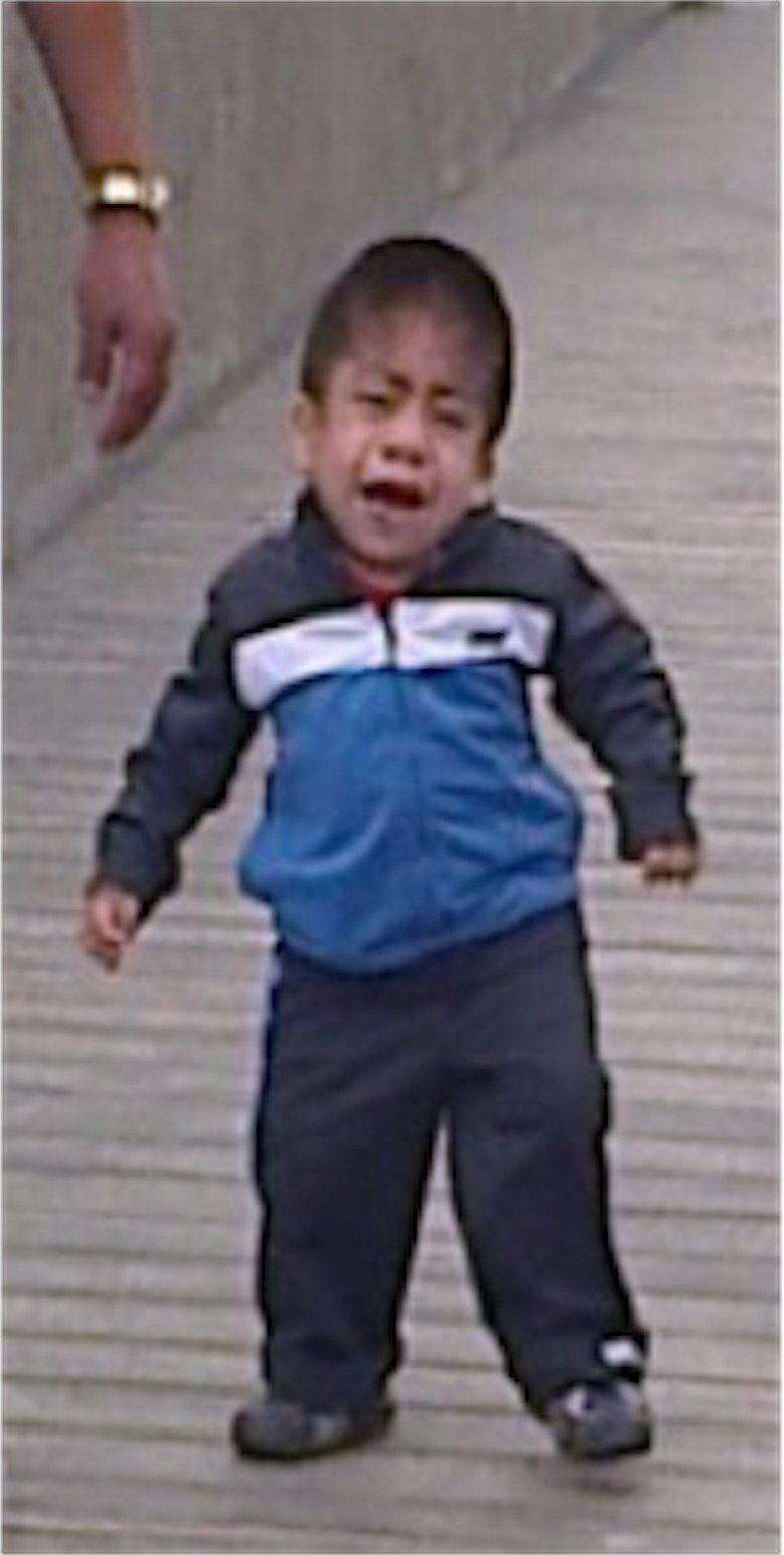
Clinical follow‐up: Patient at 3 y and 6 mo of age, showing evidence of the facial features described above, genu valgum, claw hands, and stiffness

Based on these findings, a deficiency of N‐acetylgalactosamine 4 sulfatase (MPS VI) was diagnosed and the patient was referred to the Central Military Hospital for enzyme replacement treatment with galsulfase. The physical examination showed a patient 3 years and 6 months of age with global growth and developmental delay (*z*‐score −0.88 for weight and −2.83 for height); head circumference greater than the 95th percentile; genu valgum associated with generalized joint stiffness and contractures; brachydactyly, claw hands, trigger finger, and loss of fine motor skills; corneal opacity; small and spaced‐out teeth; reducible umbilical hernia; and hepatomegaly (Figure 2).

Assessment by a multidisciplinary team found mild glaucoma, allergic rhinitis, and bilateral otitis media; carpal syndrome‐like bilateral median nerve neuropathy, predominantly of the left side; mild obstructive hypopnea; cranial hyperostosis; corrected acetabular dysplasia (history of surgically corrected coxa valga); vertebral narrowing with subluxation and kyphoscoliosis; hepatosplenomegaly; microcephaly with noncommunicating hydrocephalus; and spinal cord compression at L1‐L2 and C5‐C6.

Based on these findings, enzyme replacement therapy with galsulfase (N‐acetylgalactosamine 4 sulfatase) 1 mg/kg/week was initiated at 3 years and 6 months of age. The patient received treatment at the hospital for 3 years and 4 months, but this became a burden for the family due to the need to deal with transportation issues and long travel time in order to attend the infusion sessions. After careful consideration of the patient's characteristics, the decision was made to initiate home therapy under a protocol in which a nurse was trained to infuse the medication at home and detect, manage, and report any adverse reaction which could occur during the infusion. The patient has been receiving home therapy for 2 years and 6 months, with good tolerance and no adverse reactions reported. This treatment has enabled continuous patient adherence to the medication, with improved quality of life for both patient and family. Additionally, home therapy has resulted in a lower risk of infection during this time of the COVID‐19 pandemic.

### 2.2 Outcome and follow‐up

**FIGURE 3:**
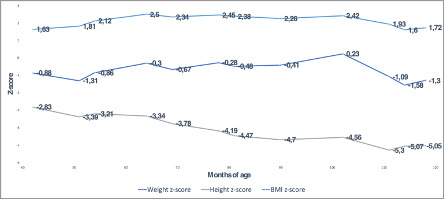
Six‐year follow‐up of weight, height, and BMI: *Z*‐score for weight, height, and BMI during patient follow‐up

Figure 3 illustrates weight, height, and body mass index (BMI) during follow‐up. During the follow‐up period, the patient was diagnosed with levocardia in situs solitus and mild aortic, mitral, and tricuspid regurgitation. Since the time of diagnosis, the progression of the cardiac compromise has been stable and slow to this date. Additionally, the patient has shown improvement in speech, motor skills, snoring, and physical endurance, enabling him to continue in school, with adequate socialization.

Glycosaminoglycan measurements in urine have remained within the expected range in accordance with the type and course of the disease on treatment, with values between 10.51 and 10.7 mg GAG/mmol/cr, slightly elevated when compared to the normal population by age (0.36‐6.36 mg GAG/mmol/cr).

**FIGURE 4:**
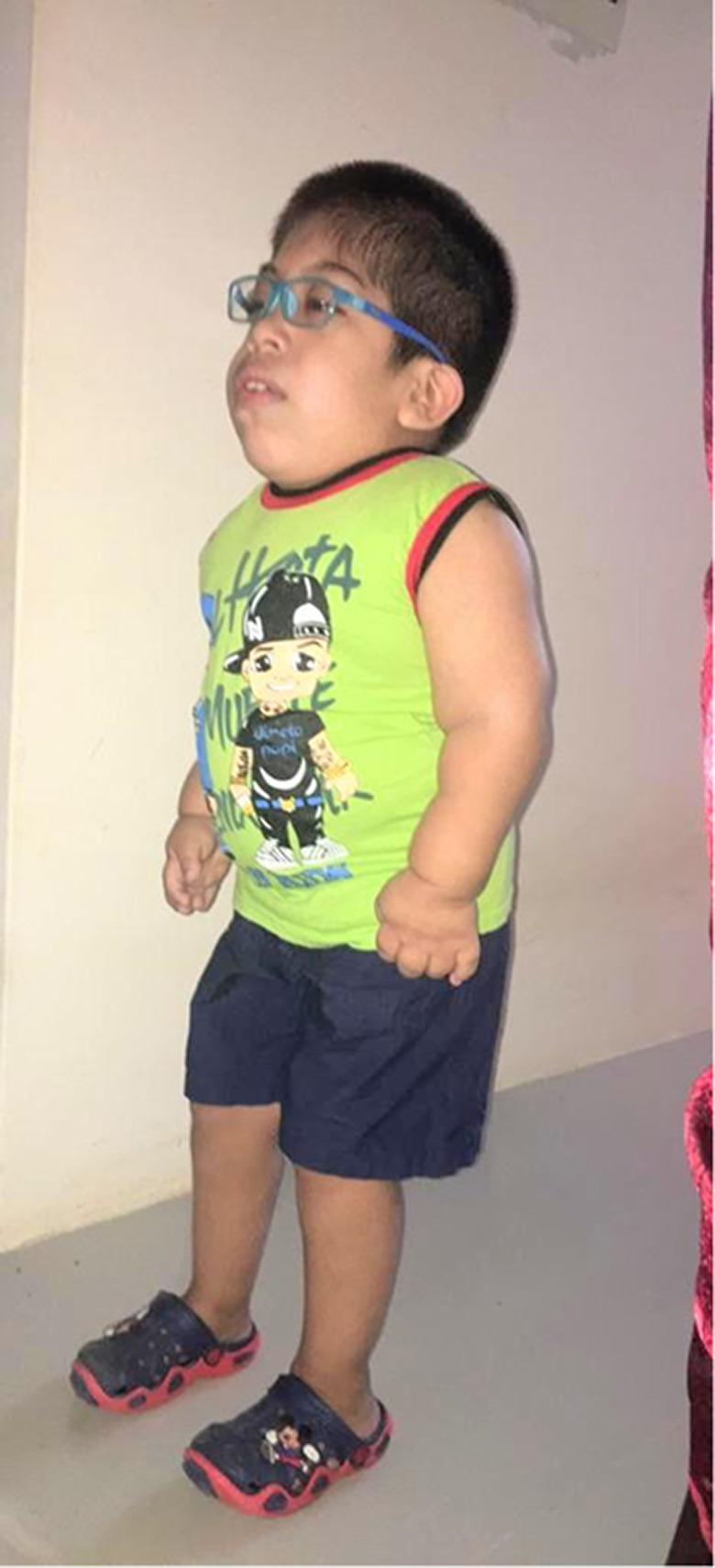
Clinical follow‐up at 9 y: Patient at 9 y of age, showing evidence of the facial features described above, lumbar spine deformation, and claw hands

No progression has been found in terms of pulmonary function tests or increased hepatomegaly or splenomegaly during follow‐up. The patient does not complain of joint pain, and reduction in generalized stiffness is evident. There is no evidence of metabolic abnormality, with glycemic and lipid profiles within normal limits. During follow‐up, the patient has undergone trabeculectomy and bilateral intraocular valve insertion for glaucoma correction, and right lower lip hemangioma resection. Auditory evoked potentials performed at 9 years of age showed severe bilateral hearing loss. Evident difficulty with activities of daily living is due to the fact that the patient is a carrier of a severe and rapidly progressive variant of MPS VI (Figure 4).

## 3 DISCUSSION

Abnormal growth is a frequent finding in MPS patients. In particular, in patients with MPS VI, normal growth is evident until 1.5 years of age and is then followed by growth stunting (−2 to −3 SD at 3.5 and 9 years, respectively).^21^ Clinical trials with ERT have shown a slight improvement in growth rate (height), which is not statistically significant when compared to patients who are not on treatment.^22^ An analysis of our patient's growth chart shows evidence of persistently low height for age (*z*‐score<−2SD) a z‐score measurement (−4.15) consistent with the mean reported by Quartel et al.^23^ and Hsiang‐Yu lin et al.^24^ for untreated patients with MPS VI. This is consistent with the limited effect on growth found in Italian and Taiwanese patient cohorts treated with ERT.^16^, ^22^ As shown in Figure 2, a progressive increase in weight (*z*‐score +1.63 to +2.4) and BMI (obesity ranges) was documented. These findings prompted probing into eating habits and physical activities, revealing a hypercaloric diet and sedentarism, the latter due in part to challenges with physical activity secondary to the disease. This explains obesity in our patient, although there is no evidence of metabolic involvement.

Among patients with MPS VI, 96% have cardiac compromise. During follow‐up, our patient was diagnosed with mild aortic, mitral, and tricuspid regurgitation. Cardiac parameters show slow progression, consistent with reports from clinical trials with ERT, with no evidence of regression of findings but rather a slow, stable course.^13^ Moreover, the patient has preserved lung function with no evident progression of the disease.

One of the most striking findings in this patient is the stabilization of urinary GAG levels. Measured levels support functional improvement with the treatment both from the pharmacodynamic and the biochemical standpoints, supporting stabilization of disease progression.^13^, ^16^

Disease stabilization and subsequent prevention of GAG accumulation have resulted in a stable cardiac and pulmonary course, as well as improvement of joint pain, fine motor skills, and speech and motor skills, enabling the child to continue in school, with adequate socialization. These are relevant outcomes derived from the use of ERT.

During the follow‐up period in clinical trials carried out in developed countries, patients on ERT are shown to have a high rate of adherence to treatment (94.6%). However, these data do not reflect what happens in real life in Latin American countries where, despite the unavailability of ERT adherence statistics, it is estimated that compliance with treatment is lower.^13^ In this patient, even in the face of challenges to ensure adequate follow‐up, the treatment has been discontinued only on rare occasions because, as a beneficiary of special insurance coverage, the patient has had access to treatment and home infusions, with ensured adherence. Home infusion has been described in developed countries as an alternative for ERT administration in patients with MPS. Nevertheless, galsulfase therapy is generally administered in a hospital setting,^18^, ^19^, ^20^ with potential negative impacts in terms of patient and family quality of life and adherence to treatment. We report the case of a patient on long‐term home therapy (to our knowledge, this is the first report of home therapy with galsulfase in Latin America), without reported side effects, with adequate treatment adherence and improved quality of life. This has been especially important during the COVID‐19 pandemic, when home therapy has ensured treatment administration during lockdowns and minimized the risk of infection.

One of the limitations of this case report has been the inability to ensure regular patient follow‐up through office visits and regular testing as planned. However, the patient has been followed in terms of clinical growth measurements, ophthalmological assessment, and cardiovascular, neurological, and endocrine function, showing limitation of disease progression and absence of drug‐related adverse events.

## 4 CONCLUSIONS

Enzyme replacement therapy is usually administered in a hospital setting. Although home infusions of galsulfase are not the standard of administration, even in some developed countries, hospital infusions imply periodic long‐term hospitalizations, affecting treatment adherence and quality of life in patients with MPS VI. We report the case of a patient who has remained on enzyme replacement therapy for more than 6 years, with home administration during the past 2 years and 6 months, adequate adherence to the treatment, and no adverse reactions. GAG levels in the urine have remained stable throughout treatment. The evolution of the disease has been slow in terms of cardiac and pulmonary involvement. Observed improvements in motor skills and pain control have enabled continued school attendance. These findings support the long‐term benefits of galsulfase‐based ERT as an option to modify the natural history of the disease, slowing its progression and positively impacting the quality of life of patients with MPS VI, especially in a setting of home infusions. Furthermore, in the context of the COVID‐19 pandemic, home treatment has provided a unique opportunity to minimize infectious risk and guarantee treatment adherence. In terms of patient safety, this is a good alternative that should be considered.

## CONFLICT OF INTEREST

The authors declare having no conflicts of interest, in accordance with ICMJE.

## AUTHOR CONTRIBUTIONS

All authors participated in the conception, design, data acquisition, manuscript writing, revision, and final approval.

## ETHICAL APPROVAL

This study was approved by the ethics committee of the Central Military Hospital (No. 1110). Informed consent was obtained from the child's parents for publication of the clinical case, laboratory results, and paraclinical tests, as well as photographs and videos of the patient.

## EDITORIAL COORDINATION

Integralis HGS (Doctors Daniel Rodríguez and María Stella Salazar).
